# Association between leucocyte telomere length and cardiovascular disease in a large general population in the United States

**DOI:** 10.1038/s41598-019-57050-1

**Published:** 2020-01-09

**Authors:** Cheng Xu, Zhiqi Wang, Xiaoqi Su, Min Da, Zhaocong Yang, Weiwei Duan, Xuming Mo

**Affiliations:** 1 Department of Cardiothoracic Surgery, Children’s Hospital of Nanjing Medical University, Nanjing, 210008 China; 2 Department of Bioinformatics, School of Biomedical Engineering and Informatics, Nanjing Medical University, Nanjing, 211166 China

**Keywords:** Cardiology, Epidemiology

## Abstract

Leucocyte telomere length (LTL) has been reported to be linked to ageing, cancer and cardiovascular disease (CVD). This study aimed to explore the association between LTL and CVD risk in a nationally representative sample of U.S. adults. Complex associations, including nonlinearity and interaction, were also examined. A total of 7,378 subjects from the National Health and Nutrition Examination Survey (NHANES) 1999–2002 were collected. Telomere length was detected from DNA samples and expressed as the mean T/S ratio (telomere repeats per single-copy gene). We performed multiple logistic regression models and interactive analysis to explore the associations between LTL and CVD risk by adjusting for potential confounders. We also performed a sensitivity analysis to investigate the robustness of our results. Among all participants, LTL was associated with the risk of CVD (OR = 0.79, 95% CI: 0.63~0.98, P = 0.033) in a linear manner rather than in a nonlinear manner (P = 0.874). Interaction effects of LTL with both education (P = 0.017) and hypertension (P = 0.007) were observed. Furthermore, using subgroup analyses, protective effects of LTL on CVD risk were found in females and in individuals who were college graduates or above, had serum cotinine >10 ng/ml, did not have hypertension, or had normal white blood cell levels. LTL is linearly inversely associated with CVD risk in the general population of the United States.

## Introduction

Telomeres are repetitive sequences of nucleotides at the ends of chromosomes that play a role in the maintenance of genic integrity. Telomeres are involved in many physiological processes^1^ such as cell senescence^2^ and endometrial regeneration^3^. Leucocyte telomere length (LTL) is the longest at birth and decreases with age^4^. Moreover, the shortening of LTL affects the progression of diseases, including ageing^5,6^, immune dysfunction^7^, post-traumatic stress disorder^8^, diabetes^9^, cardiovascular disease (CVD)^10^, and cancer^5,11^.

CVD refers to heart and blood vessel abnormalities and is one of the most common causes of noncommunicable disease mortality^12^, accounting for approximately 17.5 million deaths worldwide^13^. The United States has the largest number of CVD patients in the world^14^. Reportedly, approximately 1,500,000 U.S. adults are affected by CVD each year^15^, and millions of Americans have CVD risk factors^16^. Peng *et al*. found that longer LTL was associated with cardiovascular health (CVH) indices (i.e., a surrogate measure of CVD risk) in American Indians^17^. Similarly, Gebreab *et al*. explored the associations between LTL and CVH and observed similar results in the general U.S. population, especially in females and non-Hispanic whites^18^. Another report showed an inverse association between LTL and coronary artery calcification in African Americans^19^. A meta-analysis reported an inverse association between LTL and the risk of CVD^10^. However, among the general U.S. population, the association between LTL and the risk of CVD remains unclear. In addition, people of different ethnicities and sexes have different LTLs^20–23^, indicating that the effect of LTL on CVD risk might vary according to different subpopulations.

Using data from the National Health and Nutrition Examination Survey (NHANES) 1999–2002, our study aimed to explore the linear association between LTL and CVD risk in the general U.S. population and identify whether there is an interaction between LTL and its covariates (i.e., whether the relative risk of LTL on CVD onset might vary according to different levels of some covariates). Furthermore, a subgroup analysis was performed to discover which subgroup of covariates is associated with LTL and CVD risk. Finally, a possible nonlinear association between LTL and CVD was also investigated in the present study.

## Result

The mean and SE of LTL were presented for the total population and grouped by covariates (Table 1). The subjects were under 65 years old, had a BMI lower than 25 kg/m^2^, engaged in vigorous physical activity, had blood cholesterol levels under 240 mg/dL, did not have diabetes or hypertension, and had long LTLs. However, LTL did not vary by sex, serum cotinine level, alcohol use, or white blood cell count.

**Table 1:** Leucocyte telomere length (mean ± SE) of participants according to demographic characteristics.

	N	Mean telomere length in kb pairs (SE)	P value
Age (year)			<0.001
<65	5370	5.90 (0.04)	
> = 65	2008	5.44 (0.04)	
Sex			0.277
Male	3769	5.82 (0.03)	
Female	3609	5.84 (0.04)	
Race			0.001
Mexican American	1752	5.81 (0.04)	
Other Hispanic	385	5.97 (0.12)	
Non-Hispanic White	3753	5.80 (0.04)	
Non-Hispanic Black	1281	5.96 (0.05)	
Other Race - Including Multi-Racial	207	5.85 (0.06)	
Education level			<0.001
Less Than 9th Grade	1206	5.64 (0.04)	
9–11th Grade	1316	5.77 (0.04)	
High School Grade	1715	5.82 (0.04)	
Some College or AA degree	1795	5.88 (0.04)	
College Graduate or above	1335	5.87 (0.04)	
Serum Cotinine (ng/mL)			0.780
≤0.011 (Limit of Detection)	786	5.83 (0.06)	
0.011–10	4608	5.82 (0.04)	
>10	1881	5.84 (0.04)	
BMI (kg/m^2^)			<0.001
<25	2280	5.91 (0.04)	
25–30	2595	5.80 (0.04)	
≥30	2263	5.77 (0.04)	
Physical Activity			<0.001
None	3127	5.77 (0.04)	
Moderate	1844	5.75 (0.04)	
Vigorous	2173	5.95 (0.04)	
Alcohol Consumption			0.060
No	2173	5.79 (0.04)	
Yes	4792	5.85 (0.04)	
Energy Intake (kcal)			<0.001
<=1914	3633	5.77 (0.04)	
>1914	3455	5.88 (0.03)	
Diabetes			<0.001
No	6640	5.84 (0.04)	
Yes	725	5.65 (0.04)	
Hypertension			<0.001
No	3782	5.92 (0.04)	
Yes	3593	5.69 (0.04)	
Blood Cholesterol (mg/dL)			<0.001
<=240	6168	5.85 (0.04)	
>240	1206	5.73 (0.04)	
White Blood Cell (10^9^/L)			0.916
<4	173	5.83 (0.09)	
4–10	6620	5.83 (0.04)	
>10	580	5.82 (0.05)	

Mean (SE).

Table 2 suggests a decrease in CVD prevalence risk with each 1 kilobase pair increase in LTL (OR = 0.79, 95% CI: 0.63~0.98, P = 0.033) under a fully adjusted model (model 2). The nonlinear model did not achieve a better fit of the relationships (P = 0.874). The adjusted OR of people in the highest quartile of LTL was 0.70 (95% CI: 0.47~1.05), adjusting for age, sex, ethnicity, education level, BMI, physical activity, daily energy intake, alcohol use, serum cotinine, diabetes, hypertension, cholesterol levels, and white blood cell count and using the lowest quartile for comparison. The crude model (model 1) presented similar results. Furthermore, differences were not identified between LTL and different outcomes (congestive heart failure, coronary heart disease, angina/angina pectoris, heart attack, and stroke) after adjusting for age, sex, ethnicity, education level, BMI, physical activity, daily energy intake, alcohol use, serum cotinine, diabetes, hypertension, cholesterol levels, and white blood cell count (Supplemental Table 1).

**Table 2:** Multivariable associations of leucocyte telomere length (LTL) with cardiovascular risk in U.S. adults 1999–2002.

	Model 1		Model 2	
	OR (95% CI)	P value	OR (95% CI)	P value
LTL				
Linear model	0.77 (0.61, 0.96)	0.019	0.79 (0.63, 0.98)	0.033
Nonlinear model		0.922		0.874
LTL quartiles				
<=5.294	Ref		Ref.	
(5.294, 5.658]	1.04 (0.77, 1.42)		1.08 (0.79, 1.46)	
(5.658, 6.085]	0.82 (0.56, 1.20)		0.86 (0.58, 1.28)	
>6.085	0.66 (0.44, 1.01)		0.70 (0.47, 1.05)	

Model 1: adjusted for age, sex, race, and education levels.

Model 2: model 1 plus adjusted for cotinine, physical activity, alcohol consumption, energy intake, BMI, diabetes, hypertension, blood cholesterol, and white blood cells.

**Figure 1:**
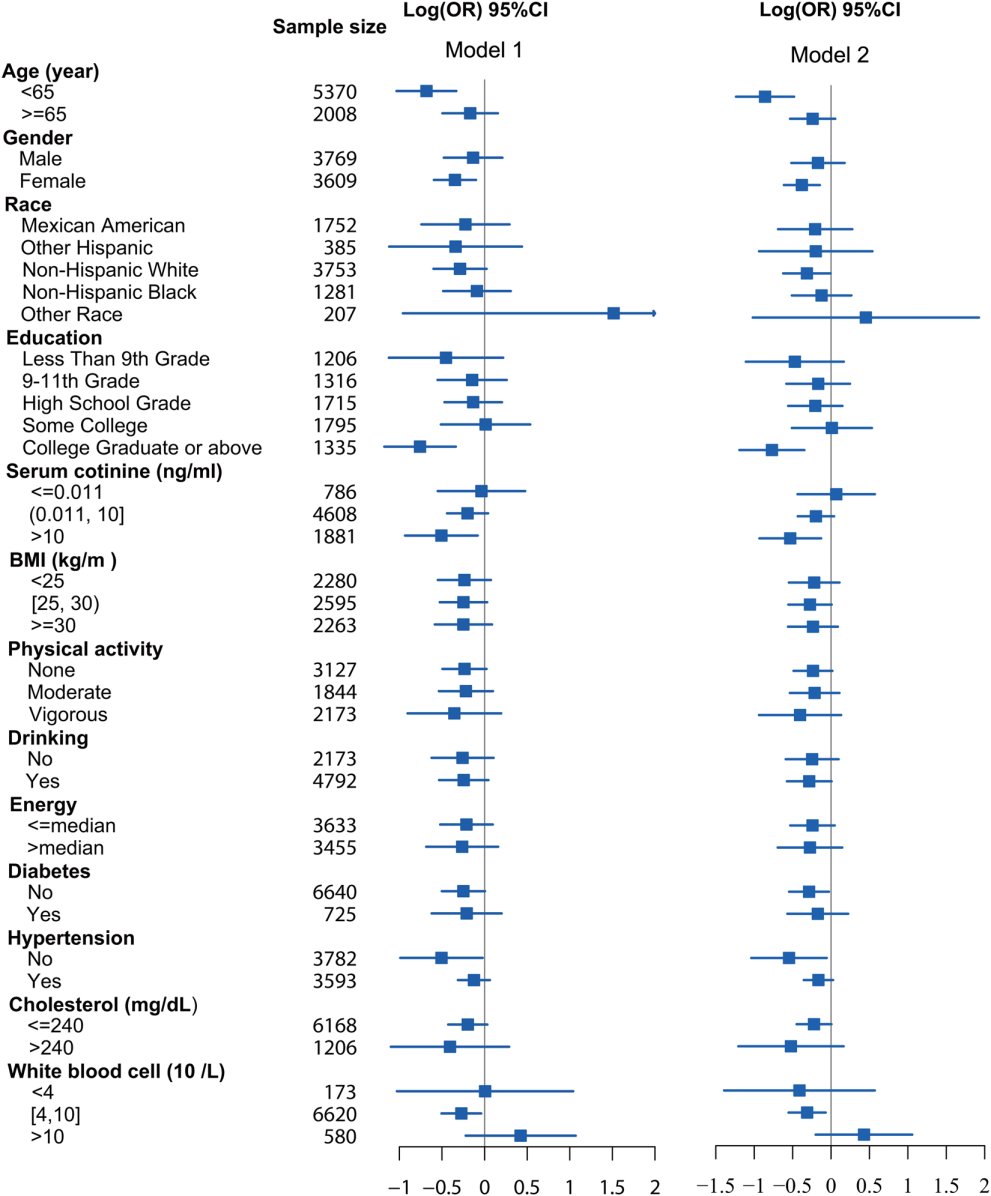
Multivariable logistic regression associations of leucocyte telomere length with CVD risk in U.S. adults 1999–2002 by subgroup analysis. Model 1: adjusted for age, sex, race, and education levels. Model 2: adjusted for age, sex, race, education levels, cotinine, physical activity, alcohol consumption, energy intake, BMI, diabetes, hypertension, blood cholesterol, and white blood cells.

**Figure 2:**
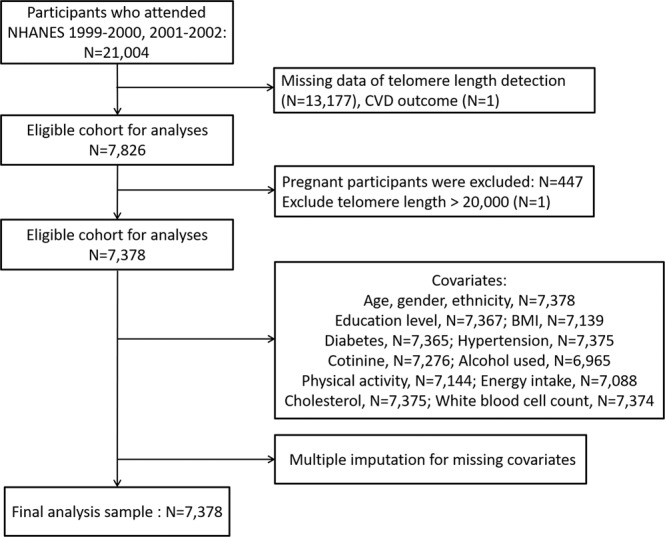
Eligible participants and those included in the analyses of the associations between leucocyte telomere length and CVD risk in adults.

Interaction analysis under model 2 revealed two interaction effects of LTL with both education levels (P = 0.017) and hypertension (P = 0.007) (Table 3). All results of hypothesis testing on interaction terms are presented. Figure 1 shows the varying log(OR) and 95% CI of LTL with different continuous covariates and subsequently helps to identify the changing trend of log(OR) as well as the beneficial subgroup of covariates. The protective effect of LTL on CVD risk shows different trends (although not statistically significant in terms of the interaction) in absolute value with age, serum cotinine, BMI, daily energy intake, cholesterol levels, and increasing white blood cell count. Moreover, this protective effect of LTL remained almost different (the dashed line did not go through the 95% confidence band) for people under age 62, with log-transformed serum cotinine >−1, BMI <30, or WBC <7. Subgroup analysis of model 2 indicated that LTL had a protective effect on CVD risk in females but not in males (Fig. 2), and younger age groups (<65) showed a stronger protective effect of LTL on CVD risk. In addition, subgroups with a college degree or above, serum cotinine levels >10 ng/ml, non-hypertension, or normal white blood cell levels (4–10 × 10^9/L^) show a protective effect of LTL on CVD risk. However, there was no difference in other covariates, including BMI, exercise, alcohol consumption, energy intake, diabetes, and cholesterol levels. The results of the crude model (model 1) were similar to those in model 2.

**Table 3:** P-value of the interaction effect of leucocyte telomere length (LTL) and each covariate on cardiovascular risk.

Interaction term	Model 1	Model 2
LTL: Age	0.112	0.212
LTL: Sex	0.529	0.391
LTL: Race	0.597	0.674
LTL: Education levels	0.006	0.017
LTL: Serum cotinine	0.127	0.159
LTL: BMI	0.554	0.466
LTL: Physical activity	0.359	0.560
LTL: Alcohol consumption	0.242	0.489
LTL: Energy intake	0.430	0.399
LTL: Diabetes	0.475	0.517
LTL: Hypertension	0.009	0.007
LTL: Blood cholesterol	0.866	0.991
LTL: White blood cells	0.029	0.142

Model 1: adjusted for age, sex, race, and education levels.

Model 2: adjusted for age, sex, race, education levels, cotinine, physical activity, alcohol consumption, energy intake, BMI, diabetes, hypertension, blood cholesterol, and white blood cells.

In sensitivity analyses, we conducted logistic regression by adjusting different covariates. The associations between LTL and CVD risk remained almost unchanged (Supplemental Table 2, Fig 1).

## Discussion

In this study, we found a negative association between LTL and CVD risk in the general population of the United States, and the trend of the overall correlation is linear rather than nonlinear. We also observed that education levels and hypertension status had interactive effects with LTL on CVD risk. Additionally, age under 65 years old, female sex, college degree or higher, higher cotinine level, normotension, and normal white blood cell levels were the suggested trends of association of LTL with CVD risk. These findings suggest that telomere length may be a more useful indicator of CVD risk for specific subpopulations.

There are many studies on telomere length and CVD risk. Most previous studies have indicated that LTL is negatively associated with CVD risk worldwide^10,24,25^. For instance, the results of a Danish study with a large sample size suggest that short telomere length is associated with only a modestly increased risk of myocardial infarction and ischaemic heart disease^26,27^. Our present study in the American population is also consistent with the above study. However, there are reports of negative results, and a study on the Canadian general population showed that LTL and incident coronary heart disease risk are not linearly associated^28^. We speculated that the inconsistent findings may be due to the different covariate corrections, e.g., smoking, alcohol consumption and energy intake. These variables related to CVD^29,30^ were not adjusted, and different genetic backgrounds may increase the risk of CVD^31^. In addition, several animal experiments have also confirmed the relationship of LTL with age-dependent cardiac disease^32^ and dilated cardiomyopathy^33^. The results of the present study in the general United States population were consistent with previous conclusions^10,24–27^. Furthermore, we also suggested a linear rather than nonlinear association between LTL and CVD risk. Thus, in the CVD field, longer LTL may be better. However, it is worth noting that longer LTL may be a likely risk factor for cancer^34^.

The occurrence and development of CVD may lead to death^35^. Shortening of the telomere length can increase CVD-related death in African-American individuals^36^. Although the same NHANES database was used, we focused on CVD risk; thus, we included people over the age of 20, and the study of the association of telomere length and mortality included subjects who were over 50 years old. Even so, it is suggested that shortening of the telomere length may have many effects and may increase the risk of CVD, leading to death. In addition, some biomarkers related to CVD are also associated with telomere length^37^. Rehkopf *et al*. found that telomere length was negatively associated with BMI, waist circumference, percentage of body fat, triglycerides, pulse rate, and cystatin C. Additionally, telomere length was positively associated with high-density lipoprotein cholesterol. Among the above biomarkers, for example, we adjusted the BMI variable as a covariate in our data analysis to improve the robustness of our results.

The effects of interactions of all included covariates with LTL on the risk of CVD were assessed, and we found that education levels and hypertension status modify its risk, while others do not. Additionally, the results of the subgroup analysis showed that LTL was associated with CVD risk in subjects with college degrees or above. We suspected that there were many reasons for this phenomenon, combining social and complex factors^38^. At the same time, our study observed that the population with college degrees or higher had lower CVD morbidity (data not shown), which is consistent with previous studies that have reported that lower education levels may contribute to CVD risk factors^39^. It is possible that more educated subjects may benefit from the increased health knowledge and a healthier life style^40,41^. Regarding hypertension, we found an inverse association between LTL and CVD risk in people without hypertension. Hypertension is a cardiovascular risk factor^42^, and our results also found an increase in the prevalence of CVD in hypertensive people (data not shown), which may affect the association. In addition, the LTL of non-hypertensive patients in the present study was longer than that of hypertensive patients, which was consistent with the results of a previous meta-analysis^43^, possibly due to the shortening of the telomere length of endothelial progenitor cells in hypertensive patients^44^. The presence of high blood pressure may have affected the association, but the specific reason is unknown.

We also found that there was a negative association between LTL and CVD risk in the population with higher cotinine (>10 ng/ml) exposure. Cotinine is a biomarker of active and passive smoking; smoking is considered a risk factor for CVD^29,45^. In addition, smoking can reduce telomere length^46^, which may explain why the association was more pronounced with high cotinine exposure. Furthermore, some studies suggest that inflammation and oxidative stress can reduce LTL and then cause CVD^6^. Smoking increases inflammation and oxidative stress^47,48^, which may be one of the causes of the above association among subjects with higher cotinine levels.

In our results, an inverse association between LTL and CVD risk was found in the female population. In our study, the LTL of females was approximately the same length as that of males, while other studies have suggested that the LTL of females is longer than that of males^49,50^. One explanation may be oestrogen^51^. The LTL of postmenopausal women after hormone therapy is longer than that of similarly aged women without hormone therapy^52^, suggesting that oestrogen may increase LTL. In addition, another possible explanation for the sex differences associated with LTL and CVD include social factors related to gender^53^, which may be the sex differences in the LTL‐CVD association.

This study has the following advantages. First, for the first time with a large sample size, we found an association between LTL and CVD risk that presented a negative linear correlation in the general population of the United States, which is of value for further biological research. Second, the subgroup analysis identified susceptible populations, and these subjects deserve more attention. Our findings provide not only clues for follow-up research but also a theoretical basis for advice and guidance on relevant prevention and treatment and the formulation of government policies.

Several limitations exist in the present study. First, this study is a cross-sectional study and cannot demonstrate a causal relationship. Although previous studies have generally assumed telomere length to be the cause^25^, strictly speaking, our study provides only an association. Second, previous studies indicated that genetic factors played a role in the occurrence of CVD^54^, but there was no relevant information in the database, so there was no way to extract and analyse it. Third, the sample size is still small, and our outcome variables were combined, so we could not analyse the effects of LTL and individual cardiovascular diseases.

## Method

### Study population

**Figure 3:**
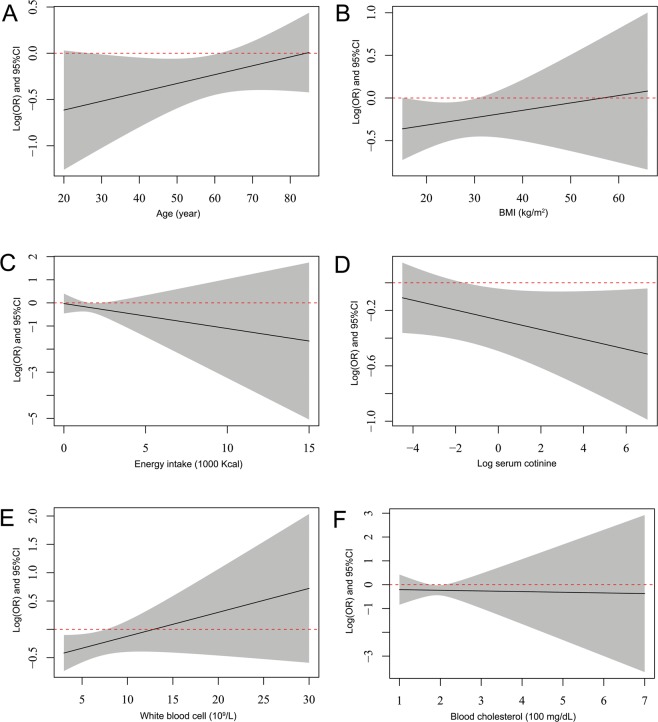
Interactions of the varying effect of LTL on CVD risk with the continuous covariates: (**A**) age, (**B**) BMI, (**C**) energy intake, (**D**) log-transformed serum cotinine, (**E**) white blood cell counts and (**F**) blood cholesterol.

The subjects were from the NHANES 1999–2000 and 2001–2002 cycles. The NHANES 1999–2000 and 2001–2002 protocols were approved by the National Center for Health Statistics Research Ethics Review Board (ERB) of the Centers for Disease Control and Prevention (Protocol #98–12). Provided sample weights in NHANES were used to account for the complex sampling of the NHANES data and reflect the non-institutionalized population of the United States. A total of 21,004 subjects were obtained from the NHANES 1999–2002. We, in sequence, excluded data from participants with pregnancy status (n = 676), those missing data for LTL detection (n = 12,948), those missing data for CVD outcome (n = 1), and LTL > 20 kilobase pairs (kbp, n = 1). The detailed flow chart is summarized in Fig. 3. All cycles of the NHANES protocols were approved by the NCHS Research Ethics Review Board^55^, and the data user agreement was online (https://www.cdc.gov/nchs/data_access/restrictions.htm). All data were retrieved from the website of the National Center for Health Statistics^56^. Written informed consent of all participants was obtained.

### CVD outcome

The CVD outcomes were assessed by a standard questionnaire survey conducted by the National Center for Health Statistics (NCHS) during a personal interview. The participants were asked to answer whether they had been diagnosed by a doctor with the following diseases: congestive heart failure, coronary heart disease, angina/angina pectoris, heart attack, and/or stroke. The sample numbers and percentages of congestive heart failure, coronary heart disease, angina/angina pectoris, heart attack, and stroke in our data were as follows: 230 (3.1%), 328 (4.5%), 278 (3.8%), 335 (4.5%), and 237 (3.2%), respectively. If one of the answers was yes, we regarded the subject as having a positive outcome. There were 821 subjects with at least one positive outcome, which accounted for 11.1% of our analysed population.

### Telomere length evaluation

The telomere length was calculated as in our previous study^57^. Briefly, DNA was extracted and purified following the Puregene kit protocol (Gentra systems, Inc., Minneapolis, Minnesota, USA), and the quantitative polymerase chain reaction method was applied to measure telomere length. The DNA source used for a reference of the T/S ratio was from the human diploid fibroblast cell line IMR90. The result of telomeric fragment length from Southern blot analysis was used to calculate and convert the T/S ratio (telomere repeats per single-copy gene) to base pairs. Then, according to the formula kbp = (3274 + 2413 * (T/S))/1000 provided by NHANES, telomere length was presented in kilobase pairs (kbp).

### Covariates

Four types (demographic, anthropometric, lifestyle, and medical comorbidities and biomarkers) of covariates were applied in the model. The demographic variables include age, sex, ethnicity, and education level. We regarded age as a binary variable of less than 65 years old and greater than or equal to 65 years old. The ethnicity variable was classified as Mexican American, Other Hispanic, Non-Hispanic White, Non-Hispanic Black, and Other Race. Less Than 9th Grade, 9–11th Grade, High School Grade, Some College or AA degree, and College Graduate or above were the five categories used to classify education level. The anthropometric variable was BMI. BMI is defined as weight (kilogram) divided by the square of height (metre). We divided BMI values (kg/m^2^) into three categories, less than 25, 25–30 and more than 30. The lifestyle variables were physical activity, daily energy intake, alcohol consumption, and smoking exposure (serum cotinine). The amount of physical activity was obtained through a questionnaire survey, which was divided into three categories: none, moderate, and vigorous. Daily energy intake was divided into less than median and more than median according to calories. According to the quantified value of alcohol intake from the dietary questionnaire, alcohol consumption was classified as non-alcohol consumption and alcohol consumption. Because there are active and second-hand exposures to smoking and the questionnaire can reflect only the situation of active smoking, we use serum cotinine to reflect smoking exposure. Those with values less than the detection limit were classified into the unexposed group, those with serum cotinine levels between the detection limit and 10 ng/ml were in the low exposure group, and those with values above 10 ng/ml were in the high exposure group. The medical comorbidities and biomarkers included diabetes, hypertension, cholesterol levels, and white blood cell count. There were three indicators in the definition of diabetes: a) whether the patient self-reported that a doctor informed him or her of a diagnosis of diabetes; b) HbA1c index ≥ 6.5%; or c) fasting blood glucose ≥ 126 mg/dL. Meeting one of the three indicators was defined as diabetes, and not meeting any of the three was defined as non-diabetes. The standard of hypertension was a) use of antihypertensive drugs, or b) a systolic blood pressure greater than or equal to 140 mmHg or a diastolic blood pressure greater than or equal to 90 mmHg. A subject meeting one of the above criteria was considered to have hypertension; otherwise, it was considered to be non-hypertension. Blood cholesterol was measured with an enzymatic method and classified into two categories, less than or equal to 240 mg/dL and more than 240 mg/dL. White blood cells were detected by the Beckman Coulter method and divided into three categories, less than 4 × 10^9^/L, 4–10 × 10^9^/L and more than 10 × 10^9^/L. All of the variables were obtained from questionnaires, laboratory test results, or physical examination results.

### Statistical method

All analyses in this study accommodated the complex sampling design and sample weights according to NHANES guidelines. We used multivariate imputation by chained equations (MICE) to impute the missing values (missing rate varying from 0~5.6%) of the covariates mentioned above to maintain statistical power. The number of multiple imputations was set to 5, and each estimator in the subsequent analysis of multiple imputed data would be further combined. The data on characteristics are presented as the mean and standard error (SE). We employed a linear regression model (being equivalent to a t test or ANOVA) to compare the distributions of LTL within subgroups of each characteristic. We then combined the estimators in regression from 5 imputations and obtained an overall estimator for each parameter. Finally, we used the Wald test to obtain the P value for each grouped characteristic. The odds ratio (OR) and 95% confidence interval (CI) showed the association between LTL and risk of CVD by using logistic regression, considering LTL as a continuous variable and as a categorical variable by quartiles. Model 1 was adjusted for age (continuous), sex, ethnicity, education level, and BMI (continuous). Model 2 was based on model 1 and physical activity, daily energy intake (continuous), alcohol use, serum cotinine (continuous, log-transformed), diabetes, hypertension, cholesterol levels (continuous), and white blood cell count (continuous). The sensitivity analysis was used to estimate the robust result by adjusting model 1 plus physical activity, daily energy intake, alcohol use, and serum cotinine. We performed restricted cubic spline regression with 3 knots located at the 10^th^, 50^th^, and 90^th^ percentiles of LTL (corresponding to 5.01, 5.66, and 6.55 kbp) to explore the underlying nonlinear association between LTL and CVD risk. Our models considered a product term between LTL and covariates to examine the interaction effects on CVD risk. Furthermore, we estimated the effect of LTL on CVD risk under a specific group of each covariate by subgroup analysis. We performed all analyses with R v3.5.0, and *P* values less than 0.05 were considered indicative of statistical significance.

## Supplementary information

Supplementary Information.
