# Supplementary material for: Association between leucocyte telomere length and cardiovascular disease in a large general population in the United States
Source: Sci Rep. 2020 Jan 9;10:80. doi: 10.1038/s41598-019-57050-1 (PMC6952450; doi:10.1038/s41598-019-57050-1)
Supplement: Supplementary file 1 — Supplementary Information. [file 41598_2019_57050_MOESM1_ESM.pdf]

Association between leucocyte telomere length and cardiovascular disease in a large general population in the United States

Cheng Xu[1,*], Zhiqi Wang[1,*], Xiaoqi Su[1,*], Min Da[1], Zhaocong Yang[1], Weiwei Duan[2,#], Xuming Mo[1,#]

[1] Department of Cardiothoracic Surgery, Children's Hospital of Nanjing Medical University, Nanjing, 210008, China.

[2] Department of Bioinformatics, School of Biomedical Engineering and Informatics, Nanjing Medical University, Nanjing, 211166, China.

*These authors contributed equally to the present study and should be regarded as joint first authors.

Running title: telomere length and cardiovascular disease

[#]Correspondence to:

Weiwei Duan, Department of Bioinformatics, School of Biomedical Engineering and Informatics, Nanjing Medical University, 101 Longmian Avenue, Nanjing, 211166, China. Tel: 86-25- 86869366. Email: passion@njmu.edu.cn

Xuming Mo, Department of Cardiothoracic Surgery, Children's Hospital of

Nanjing Medical University, 72 Guangzhou Road, Nanjing, 210008, China.

Tel: 86-25- 83117234. Email: mohsuming15@sina.com

Table S1. Multivariable associations of leucocyte telomere length (LTL) with congestive heart failure, coronary heart disease, angina/angina pectoris, heart attack, and stroke risk in U.S. adults 1999–2002.

	Congestive heart failure	Coronary heart disease	Angina/ angina pectoris	Heart attack	Stroke
Case	230	328	278	335	237
Continuous LTL	0.91 (0.68, 1.21)	1.06 (0.82, 1.37)	0.86 (0.58, 1.26)	0.78 (0.52, 1.18)	0.85 (0.59, 1.23)
LTL Quantiles					
<=5.294	Reference	Reference	Reference	Reference	Reference
5.295~5.658	1.08 (0.69, 1.70)	1.19 (0.84, 1.68)	1.32 (0.90, 1.93)	0.76 (0.50, 1.16)	1.18 (0.76, 1.83)
5.659~6.085	1.01 (0.55, 1.87)	1.05 (0.66, 1.66)	1.10 (0.67, 1.81)	0.85 (0.55, 1.31)	0.86 (0.48, 1.53)
>6.085	0.94 (0.55, 1.61)	1.27 (0.75, 2.13)	0.81 (0.37, 1.81)	0.68 (0.37, 1.28)	0.67 (0.38, 1.17)

The result was adjusted for age, sex, race, education levels, cotinine, physical activity, alcohol consumption, energy intake, BMI, diabetes, hypertension, blood cholesterol, and white blood cells.

Table S2. Multivariable associations of leucocyte telomere length (LTL) with cardiovascular risk in U.S. adults 1999–2002.

	OR (95% CI)	P value
LTL		
Linear model	0.80 (0.65,0.99)	0.043
Nonlinear model		0.834
LTL quartiles		
<=5.294	Ref	
(5.294, 5.658]	1.05 (0.78,1.42)	
(5.658, 6.085]	0.85 (0.59,1.24)	
>6.085	0.71 (0.47,1.06)	

Adjusted for age, sex, race, education levels, cotinine, physical activity, drinking, and energy intake.

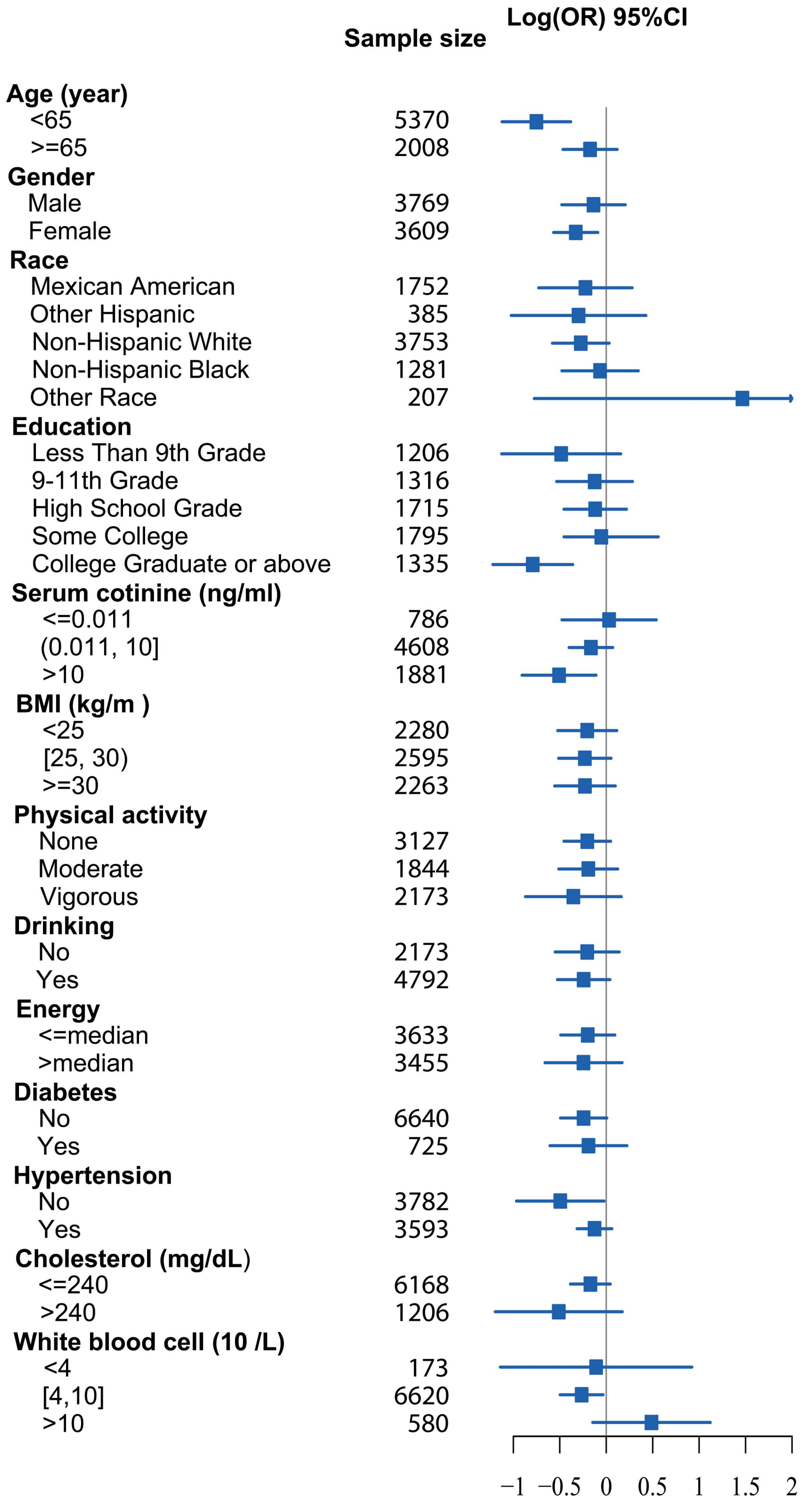
Log(OR) 95%CI
Sample size
Age (year)
<65 5370
>=65 2008
Gender
Male 3769
Female 3609
Race
Mexican American 1752
Other Hispanic 385
Non-Hispanic White 3753
Non-Hispanic Black 1281
Other Race 207
Education
Less Than 9th Grade 1206
9-11th Grade 1316
High School Grade 1715
Some College 1795
College Graduate or above 1335
Serum cotinine (ng/ml)
<=0.011 786
(0.011, 10] 4608
>10 1881
BMI (kg/m)
<25 2280
[25, 30) 2595
>=30 2263
Physical activity
None 3127
Moderate 1844
Vigorous 2173
Drinking
No 2173
Yes 4792
Energy
<=median 3633
>median 3455
Diabetes
No 6640
Yes 725
Hypertension
No 3782
Yes 3593
Cholesterol (mg/dL)
<=240 6168
>240 1206
White blood cell (10 /L)
<4 173
[4,10] 6620
>10 580
−1 −0.5 0 0.5 1 1.5 2
